# Attitudes of pregnant women regarding tobacco and alcohol use during pregnancy at one primary health care clinic in Southern Namibia

**DOI:** 10.4314/ahs.v25i2.26

**Published:** 2025-06

**Authors:** Selma Hamutenya, Emma Maano Nghitanwa, Marian Tusano Sankombo

**Affiliations:** University of Namibia, School of Nursing and Public Health

**Keywords:** Attitudes, alcohol, tobacco, pregnancy

## Abstract

**Introduction:**

Alcohol and tobacco use during pregnancy is known to negatively affect the health of the mother and the fetus.

**Objective:**

To determine the attitude of pregnant women attending antenatal care regarding the use of tobacco and alcohol during pregnancy.

**Methods:**

A descriptive, cross-sectional, analytical design was used. The population were all pregnant women attending antenatal care at the clinic where study was conducted. Systematic sampling method was used to select the sample of 224 pregnant women. Data was collected using a self-administered questionnaire. Data was analysed using Statistical Package of the Social Sciences (SPSS) version 27. Descriptive statistics was utilized to generate frequencies and percentages. Fisher's Exact test at 0.05 alpha level was used to determine the association between variables.

**Results:**

Most participants, 92 (43.6%) were aged between 18 and 24 years. Majority, 186 (88.15%) were in third trimester of pregnancy, 154 (73%) were single, and unemployed. Most participants 165 (78.2%) have positive attitude regarding the use of alcohol during pregnancy and 198 (98.3%) have positive attitude regarding tobacco use during pregnancy. Further, marital status showed a significant association with the attitudes towards alcohol use (p=0.042).

**Conclusion:**

Participants have positive attitude regarding alcohol and tobacco use during pregnancy.

## Introduction

Globally, alcohol and tobacco use during pregnancy is considered a challenge and has been linked to negative effects on a person's health and wellbeing^1^. Alcoholic beverages are described as liquids with alcohol (ethanol) in them that are meant to be consumed. The majority of the alcoholic drinks are made through the procedures of fermentation and distillation, but some are also made through the process of blending^2^. Smoking is defined as breathing in tobacco smoke from cigarettes, pipes, or cigars^3^. The Centre for Disease Control (CDC)^4^ has been advocating for pregnant women not to consume alcohol due to the serious and detrimental effects on the development of the unborn child (foetus). Baker^5^ defines attitude as a mental or neural state of readiness that has been organised by experience and has a directional or dynamic influence on the individual's response to all objects and situations with which it is associated. According to Mariental District Hospital's maternity data from January to June 2019, there were a total of 443 delveries, with 11% (47) of those being low birth weight babies^6^. Furthermore, 76 of the 443 women who gave birth claimed that they had drunk alcohol or smoked tobacco during their pregnancy^6^. About 36 % of premature babies were delivered to mothers who smoked during pregnancy, and 25 percent of premature babies were born to mothers who consumed alcohol during pregnancy^6^. Despite the higher number of alcohol and tobacco use in Mariental health district the researchers could not find a study that determine the attitude of pregnant women on tobacco and alcohol use during pregnancy.

## Background

Despite widespread knowledge that drinking alcohol during pregnancy can result in undesirable effects such as Foetal Alcohol Spectrum Disorder (FASD), one out of every ten pregnant women in underdeveloped nations is believed to drink alcohol throughout pregnancy^7^. The global prevalence of Foetal Alcohol Spectrum Disorder (FASD), which is caused by prenatal alcohol consumption in children and adolescents, is estimated to be 7.7 per 1000 people^8^. One of the key avoidable risks linked to increased maternal and foetal morbidity and mortality is smoking during pregnancy^9^. Furthermore, smoking during pregnancy is a severe public health issue because it harms the mother and the foetus, as well as increasing the chance of premature birth^10^.

Additionally, studies have shown that moms who smoke have much lower cervix immune responses and that nicotine and tobacco-specific carcinogens have been found in the cervical mucus of smokers^11^. A toxin from tobacco smoke causes placental damage and can result in abortion, placenta praevia, or placental abruption. Smoking during pregnancy increases the likelihood of respiratory problems in infants, including obesity, behavioural issues, and asthma. In Slovenia, Rozman, Mivšek, Došle and Kusterle^12^ revealed that despite knowing and being informed about all of the problems smoking poses to the pregnancy and the foetus, about 66% of women continued to smoke during pregnancy. Smoking during pregnancy has been linked to a variety of health problems in children, including asthma, recurrent otitis media, obesity, and future nicotine addiction^13^. The European region had the highest estimated prevalence of smoking during pregnancy of 8.1%, but had the lowest estimated proportion of women who smoked daily and continued to smoke daily during pregnancy, accounting for 30.6%^14^. Alcohol intake during pregnancy was high in Sub-Saharan Africa as women continued to drink during pregnancy^15^. In a national survey, pregnant women who had given birth previously or smoked were more likely to have a tolerant attitude towards alcohol consumption during pregnancy^16^. Moreover, the results of a study done in Italy regarding the main risk factors in pregnancy indicated that a large proportion of women agreed that consumption of alcohol (91.4%) and smoking (53.7%) in pregnancy could cause harm to the foetus^17^.

A study conducted in Uganda showed that slightly more than half (54.3%) of the respondents had positive attitudes by indicating that alcohol was extremely important or very important in increasing health risks to the mother. According to a study done in South Africa on participants' attitudes towards alcohol use during pregnancy, 16.5% of the participants were classified as having harmful attitudes^18^.

## Material and method

### Design

A quantitative approach with a cross-sectional descriptive and analytical design was used in this study. A questionnaire with close ended question was used to gather the data from the participants regarding alcohol and tobacco use during pregnancy.

### Population and sampling

This study was conducted at a clinic in southern region in Namibia. The study population included all the pregnant women attending antenatal care at study site during the study time. A sample size of 224 was calculated using Yamane's formula (n =_N/1+N(a)2) at a 5% margin of error. The inclusion criteria were all pregnant women who attended the antenatal care at the specified clinic aged 18 years and above. The study excluded pregnant women who were under 18 years of age. Pregnant women diagnosed with mental illnesses and pregnant women attending antenatal care at other clinics rather than the selected clinic were also excluded. A systematic sampling was used where by pregnant women were selected at equal intervals from the antenatal care register so that every second element was included in the sample size until the desired number of participants was reached.

### Data collection

Before the main study pilot study was conducted on 22 pregnant women at Maltahohe Health Centre, who met the inclusion criteria, but did not partake in the main study. Data for the pilot study was collected from subjects with the same characteristics as those under study. The respondents completed a self-administered questionnaire to assess knowledge, attitudes, and practices of pregnant women regarding tobacco and alcohol use in pregnancy. The outcome of the pilot study was that questions were well understood, and no major changes were made

Data was collected over a period of two months with a self-administered questionnaire developed by researchers in English. The tool consisted of questions regarding sociodemographic characteristics and on attitudes regarding alcohol and tobacco use during pregnancy. Data was collected in a private room and the researchers were available for clarifications of questions when needed.

Validity was ensured by giving the questionnaires to the experts in midwifery to evaluate the content of the instrument and the questionnaire was based on literature reviews and by handing the instrument to an expert in the field to check if the instrument appears to measure what it is supposed to measure. Reliability was ensured by pilot testing of the questionnaire on 22 pregnant women prior to the actual study to ensure that all important areas of concern are reflected in it. No adjustments were done on the questionnaire after the pilot study. Furthermore, the data from all participants was collected using the same instrument with the same questions.

### Data analysis

Data were analysed using the Statistical Package for the Social Sciences (SPSS) version 26. Descriptive univariate analysis was performed for each variable, generating frequencies and percentages for sociodemographic factors. Fisher's Exact test at 0.05 alpha level was used to determine the association between sociodemographic characteristics and the level of attitude regarding alcohol and tobacco use during pregnancy.

### Ethical considerations

Ethical approval for the study was granted by the University of Namibia, Research Ethical Committee (SON/594/2020). Approval was also obtained from the Ministry of Health and Social Service Research Ethics Committee and from the regional management team and the management of the clinic. Privacy was maintained throughout the study by collecting data in a private room. Anonymity and confidentiality were ensured by not recording the participants names on the questionnaire, but to use pseudo numbers instead. The researchers made sure that the questions were well structured to prevent psychological harm to the participants by ensure that the data collection tool was validated by two senior midwives who are expert in the field. Justice was ensured by selecting participants for reasons directly related to the research problem.

## Results

### Sociodemographic characteristics

The mean age was 28.8 with a standard deviation of 6.9 years. The participants' ages were further categorized into age groups as shown in Table 1. Most participants, 92 (43.6%) were aged between 18 and 24 years. The minimum number of children for the participants was one and the maximum was eight children's. The number of children was further grouped into three categories. Most of the participants 163 (77.3%) had 0 to 2 children, 40 (19.0 % ) had 3 to 5 children while 8 (3.8%) participants had 6 to 8 children before the current pregnancy. Majority of the participants 186 (88.15%) were single, 21 (9.9% ) were married while only 4 (1.90%) had divorced. Most of the participants 154 (73%) were unemployed while 57 (27%) where employed. Figure 1 shows that most participants 153 (72.86%) had attended secondary education as their highest level of qualification, while nine (4.29%) have never attended school.

**Table 1:** Age classification of the participants

Age category	Frequency	Percentage (%)
18 - 24 Years	92	43.6
25 - 34 Years	77	36.5
35 - 44 Years	30	14.2
No age indicated	12	5.7
**Total**	211	100.0

### Attitudes on alcohol use in pregnancy

To determine participants' attitudes towards the use of alcohol in pregnancy, participants were asked six questions scaling at a three-point Likert scale. A majority of the participants 133 (63%) agreed that it is shameful for a pregnant woman to drink alcohol. A large number of participants (84.8%) agreed that it is advisable for pregnant women to stop drinking alcohol. About 164 of the participants (77.7%) believe that most pregnant women are aware of the effects that alcohol can have on the unborn child. Most participants 174 (82.5%) felt that health care workers are responsible for giving health education on the effects of alcohol use in pregnancy. These results are shown below in Table 2.

**Table 2:** Attitudes on alcohol use in pregnancy

No	Statement	Disagree	Not sure	Agree
1.	I think it is not permissible for a pregnant woman to be intoxicated.	19 (9.0 % )	67 (31.8%)	125 (59.2%)
2.	I believe it is safe to take at least one or two glasses of alcohol daily when you are pregnant.	86 (40.8 %)	46 (21.8 %)	79 (37.4%)
3.	I feel it is shameful for a pregnant woman to drink alcohol.	44 (20.9 %)	34 (16.1 %)	133 (63.0 %)
4.	I believe it is advisable for pregnant women to stop drinking alcohol.	10 (4.7 %)	22 (10.4 %)	179 (84.8 %)
5.	I believe most pregnant women are aware of the effects that alcohol can have on the unborn child.	12 (5.7 %)	35 (16.6% )	164 (77.7 %)
6.	I feel that health care workers are responsible to give health education on the effects of alcohol use in pregnancy.	9 (4.3 %)	27 (12.8%)	174 (82.5 %)

Level of Attitude towards alcohol use during pregnancy All statements on attitudes towards alcohol use during pregnancy were scored and added together to produce a total score range of six to 18. The individual scores were then categorized as follows: 6 - 12 negative attitude and 13 - 18 positive attitude as displayed in table 3.

**Table 3:** Levels of attitudes towards alcohol use during pregnancy

Attitude levels	Frequency	Percentage (%)
Negative	46	21.8
Positive	165	78.2
Total	211	100.0

### Attitudes on tobacco use in pregnancy

Participants' attitudes on the use of tobacco in pregnancy were assessed using a three-point Likert scale ranging from disagree, not sure or agree as shown in table 4. A large number of participants 172 (81.5%) agreed that it is not acceptable for a pregnant woman to smoke cigarettes. A majority of the participants 177 (83.9%) believed that it is not permissible for pregnant women to use any other forms of tobacco. Most participants 184 (87.2%) believed that smoking tobacco while pregnant can harm the unborn baby's health. Eighty-five percent of the participants (180) felt that it is the responsibility of health care workers to advise pregnant womn to stop smoking. Many participants 181 (85.8%) agreed that smoking should be avoided during pregnancy.

**Table 4:** Association of levels of attitudes towards alcohol use during pregnancy with demographic characteristics

Demographics Characteristics		Levels of Attitudes towards alcohol use during pregnancy		p-value
		Negative	Positive	
**Mother Age**	18 – 24 years	21 (22.8%)	71 (77.2%)	0.248
	25 – 34 years	19 (24.7%)	58 (75.3%)	
	35 – 44 years	3 (10.0%)	27 (90.0%)	
**Number of children**	0 – 2	37 (22.7%)	126 (77.3%)	0.397
	3 – 5	9 (22.5%)	31 (77.5%)	
	6 – 8	0 (0.0%)	8 (100.0%)	
**Age of pregnancy**	1^st^ trimester	2 (13.3%)	13 (86.7%)	0.745
	2^nd^ trimester	14 (20.3%)	55 (79.7%)	
	3^rd^ trimester	29 (23.0%)	97 (77.0%)	
**Marital status**	Single	40 (21.5%)	146 (78.5%)	0.042
	Married	3 (14.3%)	18 (85.7%)	
	Divorced	3 (75.0%)	1 (25.0%)	
**Employment Status**	Employed	11 (19.3%)	46 (80.7%)	0.708
	Not employed	35 (22.7%)	119 (77.3%)	
**Education level**	No education	5 (55.6%)	4 (44.4%)	0.071
	Primary education	5 (21.7%)	18 (78.3%)	
	Secondary education	29 (19.0%)	124 (81.0%)	
	Tertiary education	7 (28.0%)	18 (72.0%)	

Association of levels of attitudes towards alcohol use during pregnancy with demographic characteristics

The overall levels of attitudes towards alcohol use/consumption during pregnancy was analysed for its association with demographic characteristics. The level of association and its significance were tested using Fisher's Exact test at 0.05 alpha level. Only marital status showed a significant association with the attitudes towards alcohol use (p=0.042). All the other demographic variables were not a significant association as shown in Table 5.

**Table 5:** Attitudes on tobacco use in pregnancy

No	Statement	Disagree	Not sure	Agree
1.	I think it is not acceptable for a pregnant woman to smoke cigarette.	15 (7.1 %)	24 (11.4 % )	172 (81.5%)
2.	I believe It is not permissible for pregnant women to use any other forms of tobacco.	9 (4.3%)	25 (11.8%)	177 (83.9%)
3.	I think that tobacco smoking puts you at risk of drinking alcohol while pregnant.	14 (6.6%)	49 (23.2%)	148 (70.1%)
4.	I believe smoking tobacco while pregnant can harm the unborn baby's health.	3 (1.4%)	24 (11.4%)	184 (87.2%)
5.	I believe that nurses provide pregnant women with information on smoking during pregnancy during antenatal visits.	11 (5.2%)	41 (19.4%)	159 (75.4%)
6.	I feel that it is the responsibility of health care workers to advise pregnant women to stop smoking.	13 (6.2%)	18 (8.5%)	180 (85.3%)
7.	I believe the harms of smoking tobacco in pregnancy are less compared to other risks that pregnant women face.	30 (14.2%)	78 (37.0%)	103 (48.8%)
8.	I think it is harder to quit smoking when pregnant than any other times.	39 (18.5%)	70 (33.2%)	102 (48.3%)
9.	I think smoking tobacco should be avoided during pregnancy.	10 (4.7%)	20 (9.5%)	181 (85.8%)

### Attitudes towards tobacco use during pregnancy

All statements on attitudes towards smoking during pregnancy were scored and added together to produce a total score range of 9 to 27. The individual scores were then categorized as follows: 9-18 scored negative attitude and 19 - 27 scored positive attitude. Majority 93.8 % of participants have positive attitude towards tobacco use during pregnancy.

## Discussion

The study found that majority of pregnant women were 18 to 44 years that reflect middle age which is the reproductive age^19^. The study also found that single women were more represented than married women. This contradict the study conducted in Ethiopia that found that unmarried women had poor utilization of ANC services because they were afraid to be seen pregnant^20^. The findings of the study revealed that 63% of participants agreed that it is shameful for pregnant women to drink alcohol. These findings imply that pregnant women should be aware of the effects of drinking alcohol and should consider the effects of consuming alcohol during pregnancy. A pregnant woman drinking alcohol is thus viewed as doing something shameful, as drinking is seen as endangering the unborn child and raising concerns about the mother's care.

The findings of the study revealed that 77.7% of the participants believed that most pregnant women are aware of the effects that alcohol can have on the unborn child, with 84% of the participants agreeing that it is advisable for pregnant women to stop drinking. These findings imply that expectant mothers fully recognised the risks associated with alcohol use, and it is recommended that the Mariental clinic continue to teach these lessons in order to raise community awareness. Additionally, these findings imply that pregnant women attended ANC sessions where health hazards during pregnancy were discussed. In support, Apophia^21^ revealed that the majority of participants in his study understood the effects of alcohol consumption during pregnancy, such as Foetal Alcohol Spectrum Disorders (FASD). Women who drink while pregnant put their babies at risk for FASD, as well as other negative pregnancy outcomes like stillbirth, abortion, early birth, and low birth weight new-borns^22^.

Similarly, Agiresaasi et al.^23^ reported that more than half of the respondents had positive attitudes by indicating that alcohol intake during pregnancy increase health risks to the mother and baby (54.3%). Few respondents had fair attitudes towards maternal alcohol use by indicating that alcohol was moderately important in increasing health risks to the mother and baby (13.8%). These women were aware of the negative consequences of alcohol, but their attitude towards drinking was that there was an acceptable level of alcohol that could be drank during pregnancy.

The study revealed that 78.2% had positive attitudes towards alcohol use while 21.8% had negative attitudes towards the use of alcohol. Furthermore, participants with negative attitudes may be because of resistance to change or maybe influenced by family backgrounds on the consumption of alcohol hence the continuation of drinking alcohol even after they have received teachings on the dangers of alcohol. These study results are contradictory to the study of Howlett et al.,^24^ who reported that 70% of participants in his study continued to drink alcohol while pregnant. These women were aware of the consequences of alcohol, but their attitude towards drinking was that there was an acceptable level of alcohol that could be drank during pregnancy.

The findings of the study revealed that 81.5% of the participants agreed that it is not acceptable for a pregnant woman to smoke cigarettes, with 83.9% of the participants believing that it is not permissible for pregnant women to use any form of tobacco. These findings imply that women who are pregnant have developed a negative attitude towards smoking tobacco as a result of the knowledge they learned from nurses, which includes information about the effects of substances like nicotine found in tobacco on the baby, that may lead to preterm births. In support, Dendir and Deyessa^25^ revealed that pregnant women who used tobacco while pregnant were more likely to give birth to low weight babies.

Furthermore, the findings of the study revealed that 85.3% of the participants felt that it is the responsibility of health care workers to advise pregnant women to stop smoking. These findings suggest that health professionals should always play a proactive role in teaching expectant mothers as mothers seem to respect the knowledge they receive from health experts.

The study revealed that 85.8% of the participants agreed that smoking should be avoided during pregnancy, with 87.2% of the participants believing that smoking tobacco while pregnant can harm the unborn baby's health. These findings tend to indicate that smoking has detrimental health impacts on both the mother and the unborn child as well, and participants' indications of avoiding smoking reflect their awareness of these implications. In support of the above results Esposito et al. (2015) revealed that 53.7% of women in their study agreed that smoking in pregnancy could cause harm to the foetus. Furthermore, Gorniaczyk et al.^26^ also revealed that smoking was believed to be harmful to the developing foetus by 99% of the respondents, with 57% having received that information from their doctor.

The study revealed that 93.8% of participants had positive attitudes towards tobacco use during pregnancy while 6.2% had a negative attitude. This implies that the risks associated with smoking tobacco during pregnancy was understood by the majority of participants, hence their refrain from smoking tobacco. This protects the unborn baby and the mother from health risks during pregnancy. Contrary to the study results, Casiro found that pregnant women were satisfied with the treatment they received, but that their understanding of the consequences of smoking and alcohol consumption was limited, and that their knowledge was sometimes erroneous or confusing.

## Conclusions

The study concludes that while 21.8% of respondents had a negative attitude regarding drinking alcohol, 78.2% had a good attitude. The study concludes that the highest level on attitude 84.8% was on pregnant women to stop drinking alcohol. The study concluded that women were aware of the harm alcohol can do to an unborn child, and that it is best for women to abstain from alcohol while pregnant. Furthermore, a significant portion of participants confirmed getting the information from health professionals, and that it is the health professionals' responsibility to advise women on the dangers of drinking. The study also found that 93.3% of the participants had a negative attitude towards smoking, while only 6.2% had a negative one. Furthermore, the study concluded that 87.2% of the participating women believed that smoking tobacco while pregnant can harm the unborn baby's health, that it was unacceptable for pregnant women to smoke, that smoking should be avoided, and that it may harm the unborn child. It was also determined that health professionals should educate pregnant women on the benefits of quitting smoking.

## Figures and Tables

**Table 6:** Levels of attitudes towards tobacco use in pregnancy

Attitude level	Frequency	Percentage (%)
Negative	13	6.2
Positive	198	93.8
Total	211	100.0
